# High Mobility Group Box Protein 1 Serves as a Potential Prognostic Marker of Lung Cancer and Promotes Its Invasion and Metastasis by Matrix Metalloproteinase-2 in a Nuclear Factor-*κ*B-Dependent Manner

**DOI:** 10.1155/2018/3453706

**Published:** 2018-04-19

**Authors:** Xiaojin Wu, Weitao Wang, Yuanyuan Chen, Xiangqun Liu, Jindong Wang, Xiaobin Qin, Dawei Yuan, Tao Yu, Guangxia Chen, Yanyan Mi, Jie Mou, Jinpeng Cui, Ankang Hu, Yunxiang E, Dongsheng Pei

**Affiliations:** ^1^Department of Radiation Oncology, The First People's Hospital of Xuzhou, Xuzhou, Jiangsu 221002, China; ^2^Geneis Beijing Co., Ltd., Beijing 100102, China; ^3^Department of Respiratory Diseases, The First People's Hospital of Xuzhou, Xuzhou, Jiangsu 221002, China; ^4^Department of Chest Surgery, The First People's Hospital of Xuzhou, Xuzhou, Jiangsu 221002, China; ^5^Department of Tumor, The First People's Hospital of Xuzhou, Xuzhou, Jiangsu 221002, China; ^6^Department of Gastroenterology, The First People's Hospital of Xuzhou, Xuzhou, Jiangsu 221002, China; ^7^Department of Pharmacy, Xuzhou Medical College, Xuzhou, Jiangsu 221004, China; ^8^Clinical Laboratory of Yantaishan Hospital, No. 91, Jiefang Road, Yantai, Shandong 264001, China; ^9^Laboratory Animal Center, Xuzhou Medical College, Xuzhou, Jiangsu 221004, China; ^10^Department of Pathology, Xuzhou Medical College, Xuzhou, Jiangsu 221004, China

## Abstract

Several studies have reported a significant role of high mobility group box protein 1 (HMGB1) in lung cancer. Nevertheless, there is a lack of knowledge regarding the expression of HMGB1 and its correlation with the clinicopathological features of lung cancer. In addition, the potential molecular mechanisms underlying the role of HMGB1 in lung cancer are still unknown. We therefore investigated the clinicopathological and prognostic significance as well as the potential role of HMGB1 in the development and progression of lung cancer. HMGB1 expression in the tumor tissues of the cohort correlated with clinicopathological features. Moreover, lung cell migration and invasion were significantly increased after treatment with HMGB1. The matrix metalloproteinase-2 (MMP-2) expression and activity were upregulated after treatment with HMGB1, while the upregulated expression of MMP-2 stimulated by HMGB1 in lung cancer cells was significantly reduced with the blockage of si-p65. These results indicated that HMGB1 expression was significantly associated with lung cancer progression. We also showed that HMGB1 promoted lung cancer invasion and metastasis by upregulating the expression and activity of MMP-2 in an NF-*κ*B-dependent manner. Taken together, these data suggested that HMGB1 may be a potential prognosis and therapeutic marker for lung cancer.

## 1. Introduction

Lung cancer has currently become one of the most serious diseases threatening human health. Its incidence and mortality rates are the highest of all malignant tumors in the world [1–4]. Non-small cell lung cancer (NSCLC) accounts for 80%–85% of all lung cancers that involve a multifactorial, multistep, and multistage complex process [5–9]. Although significant progress has been made in clinical treatments with the rapid development of molecular biology techniques and clinical treatments, the survival of lung cancer patients is still limited. It is therefore important to investigate the molecular mechanisms of invasion and metastasis of lung cancer and to identify potential prognostic markers.

High mobility group box protein 1 (HMGB1) is a nonhistone, chromatin-binding nuclear protein that is widely found in eukaryotic cells, with a protein structure containing two homologous DNA-binding domains (A and B boxes) and a C-terminal tail that participates in transcription, DNA preparation, cell growth and differentiation, and extracellular signal transduction [10–12]. HMGB1 has been reported to be involved in cancer development, invasion, and metastases. In addition, HMGB1 is frequently highly expressed in various malignant tumors and is an early marker for these tumors [13–17]. In recent years, various studies to characterize the expression of HMGB1 in NSCLC patients have suggested that HMGB1 plays a vital role in the diagnosis and prognosis of NSCLC [12, 18–24]. However, this suggestion is still controversial. Shen et al. [22] suggested that HMGB1 is downregulated in NSCLC tissues, while other studies reported that the level of HMGB1 is upregulated in NSCLC tissues compared with that of normal tissues [12, 20, 24]. Thus, further research is needed to clarify the possible clinical diagnostic and prognostic values of HMGB1 in NSCLC.

It is well-known that invasion and metastasis, which are the main causes of high mortality, are common characteristics of malignant tumors. Extracellular HMGB1 is thought to induce cancer cell growth, mobility, invasion, and metastasis via binding to specific membrane receptors, including the receptor for advanced glycation end products (RAGE), and blockage of the RAGE-HMGB1 complex suppresses tumor growth and metastasis [20, 25–27]. Taguchi et al. also reported that the metastasis of Lewis lung tumor cells was inhibited when treated with anti-HMGB1 antibody, suggesting that HMGB1 is associated with tumor invasion and metastasis [26].

Matrix metalloproteinase 2 (MMP2) is a member of the Ca^2+^- and Zn^2+^-dependent endogenous protease family (MMPs), whose expression in tumors is reported to be correlated with carcinoma invasion and metastasis [28–30]. MMP2 expression is upregulated in many cancers such as in glioblastomas, melanomas, breast cancers, and colon cancers [30]. It has also been reported that the high MMP2 expression in NSCLC is an independent prognostic factor that is closely related to its clinical stage, pathological grade, lymphatic metastasis, and prognosis [19].

As a key transcription factor, the nuclear factor (NF)-*κ*B plays an important role in tumor development and progression [31]. Activated NF-*κ*B can trigger chemokine and cytokine production, which further results in tissue damage [32]. Moreover, activated NF-*κ*B signaling pathways have been correlated with MMP2 expression, and there is evidence that deacetylation of NF-*κ*B reduces MMP2 expression, leading to the inhibition of NSCLC cell invasion [33].

However, the underlying mechanisms involving HMGB1 promotion of lung cancer invasion and metastasis still remain unclear and require further elucidation. In the present study, our aim was to characterize the association between the expression of HMGB1 and the clinicopathological and prognostic factors of NSCLC and to investigate how HMGB1 promotes lung cancer invasion and metastasis.

## 2. Materials and Methods

### 2.1. Materials

The lung cancer tissue chip was purchased from Shanghai core Biological Technology Co., Ltd., number HLug-Ade180Sur-01. In 90 cases of lung cancer tissues and 90 cases of lung adjacent noncancerous tissues, TNM stage was I–III stages; lymph node metastasis was N0 in 39 cases, N1–N3 in 36 cases, and N × 15 cases in other cases. The clinical data and follow-up data were complete. The operation time was 2004.7–2009.6; the follow-up time was 2014.8, followed up for 5–10 years.

A549 and H1299 cell lines were purchased from the US standard library (American Type Culture Collection, ATCC).

### 2.2. Immunohistochemical Staining

The immunohistochemical staining method we used was Envision™, performed as described previously [34]. As for tissue immunohistochemistry staining results analysis, lung cancer tissue staining results were evaluated by IRS score under double-blind conditions by two senior pathological experts. The average staining score was calculated by combining the positive staining intensity and the percentage of positive cells. Dyeing strength was as follows: nonstaining, 0 points; weak coloring, 1 points; medium coloring, 2 points; strong coloring, 3 points. The percentage of positive cells ranged from 0 to 25%, 1 points; 26% to 50%, 2 points; 51% to 75%, 3 points; 76% to 100%, 4 points. Two points multiplied by the following scores: 0 for negative; 1–3 for weak positive; 4–6 for medium positive; and 8–12 for strong positive. We stipulated 4 points and below for HMGB1 low expression and 4 points above for HMGB1 high expression.

### 2.3. In Vitro Invasion Assay

The in vitro invasion assays were carried out using Transwell® chamber with 6.5 mm diameter polycarbonate filters as described previously [35]. HMGB1 pretreated lung cancer cells digested by trypsin were suspended by serum-free medium and then counted. The concentrations of A549 and H1299 were 4.5 × 10^4^/ml and 3 × 10^4^/ml, respectively. 10% serum was added to the bottom of the 24-orifice plate and cell suspension was added to the incubation chamber for 24 hours. We made the methanol fixation at RT for 30 min and then the PBS wash twice. Crystal violet was dyed for 15~30 minutes and 5 pictures were taken at random under the microscope and counted.

### 2.4. Gelatin Zymography

Gelatin zymography was performed as described previously [36]. We collected the supernatant of HMGB1 pretreated or not lung cancer cells, measured the proteins concentration, and then mixed it with 4x loading buffer about 30–50 ug/40 ul. SDS-PAGE electrophoresis was performed at 100 V for about 1.5 hours. Low speed oscillation elution was carried out in 2.5% Triton X-100 eluted with SDS (elution for 90 minutes), followed by rinsing with distilled water and then gel incubation at 37° for 60 h until a transparent strip appears. This was then stained at RT for 1–4 h and then destained for 2–5 h to observe the MMP2 strip.

### 2.5. Western Blot Analysis

Protein level was detected by western blot analysis as described previously [37]. After treatment with HMGB-1, A549 and H1299 cells were washed twice by ice-cold PBS. Cells were lysed by RIPA lysis buffer and then proteins were extracted. Denaturation of equal amount of supernatant protein in boiling SDS sample buffer was performed and then the samples were subjected to 10% SDS-PAGE. After that, polyvinylidene difluoride membranes were used for the proteins transfer. 5% dry skim milk was used for membrane blocking and then membranes were incubated with primary antibodies p65, MMP2. In this experiment, *β*-actin was used as an internal reference for the reliability of the experiment. Finally, membranes were treated with enhanced chemiluminescent system for visualization of the protein bands. The bands were quantified using Image J software.

### 2.6. Statistical Analysis

The correlation analysis of the expression level of HMGB1 and various clinicopathologic parameters was made using Fisher exact test. The survival data were analyzed using Kaplan–Meier survival curve analysis and Log-rank test. Measurement data represents mean ± standard deviation (*x* ± *s*). The means of two groups were compared with *t* test. We used SPSS18.0 statistical software for data processing. *P* < 0.05 shows that the difference was statistically significant, *P* < 0.01 shows that there is a significant difference.

## 3. Results

### 3.1. Expression of HMGB1 in Lung Cancer Tissues and Normal Tissues Adjacent to the Lung Cancer

Immunohistochemical staining showed that the HMGB1 high expression percentages in lung cancer tissues and normal tissues adjacent to the lung cancer tissues from 90 patients were 56.7% and 30.0%, respectively, which showed that the level of HMGB1 expression in lung cancer tissue was significantly higher than in the tissue adjacent to the lung cancers (Table 1).

### 3.2. The Relationship between the Expression of HMGB1 in Lung Cancer Tissue and Clinical Pathological Parameters

HMGB1 expression level was associated with the clinical pathological characteristics of the patients with lung cancer, such as T stage (*P* = 0.027) and lymph node metastasis of the tumor (*P* = 0.019), and was closely related to the clinical stage (*P* = 0.012). However, when compared with the patients' gender, age, and size, there was no significant difference (*P* > 0.05) (Table 2).

### 3.3. The Relationship between the Level of HMGB1 Expression of Lung Cancer Tissue and the Prognoses of Patients

**Figure 1:**
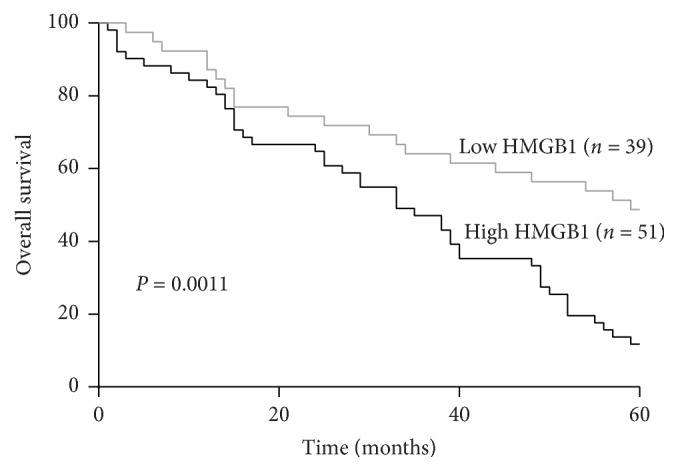
High mobility group box protein 1 expression correlated with a poorer 5-year survival for 90 lung cancer patients (*P = 0.0011)*. Significant differences were assessed by the Student *t*-test.

In order to investigate whether the high expression of HMGB1 was correlated with the poor prognosis of patients with lung cancers, we analyzed the 5-year survival rates of 90 cases of lung cancer patients using Kaplan–Meier survival curves. According to the staining intensity, we chose different time nodes using the survival analyses, which showed that the postoperative total survival percentage of high HMGB1 expression was significantly lower than low HMGB1 expression for lung cancer patients (*P* = 0.0011, HR = 0.0011, 95% CI: 0.2623 0.7166; Figure 1). Overall, the results showed that the high expression of HMGB1 was closely related to the poor prognoses of patients with lung cancer.

### 3.4. HMGB1 Promotes Lung Cancer Cell Invasion

**Figure 2:**
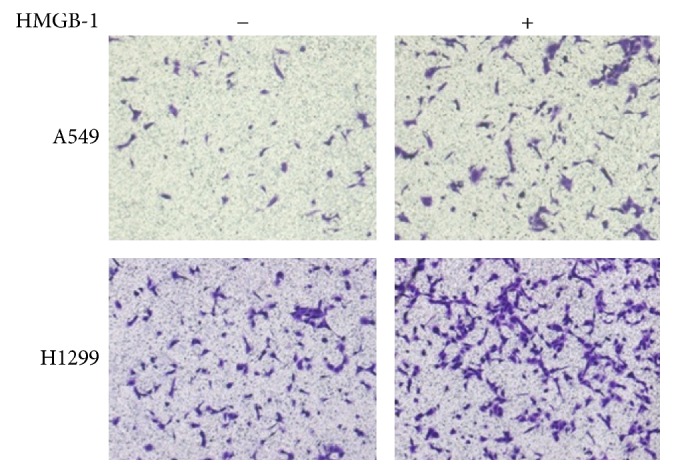
High mobility group box protein 1 (HMCB1) promoted invasion of lung cancer cells. Pretreatment of HMGB1 significantly promoted invasion of A549 and H1299 cells.

In order to determine whether HMGB1 promoted lung cancer cell invasion, the lung cancer cell lines, A549 and H1299, were pretreated with HMGB1, and the Transwell assay was used to determine the changes of cell invasion. Compared with the control group, the number of A549 and H1299 cells passing through the Transwell chambers increased by 46% and 41%, respectively, after pretreatment with HMGB1. The increased invasiveness was significantly different, suggesting that treatment with HMGB1 increases the invasive ability of lung cancer cells (Figure 2).

### 3.5. HMGB1 Activates MMP2 Enzyme Activity

**Figure 3:**
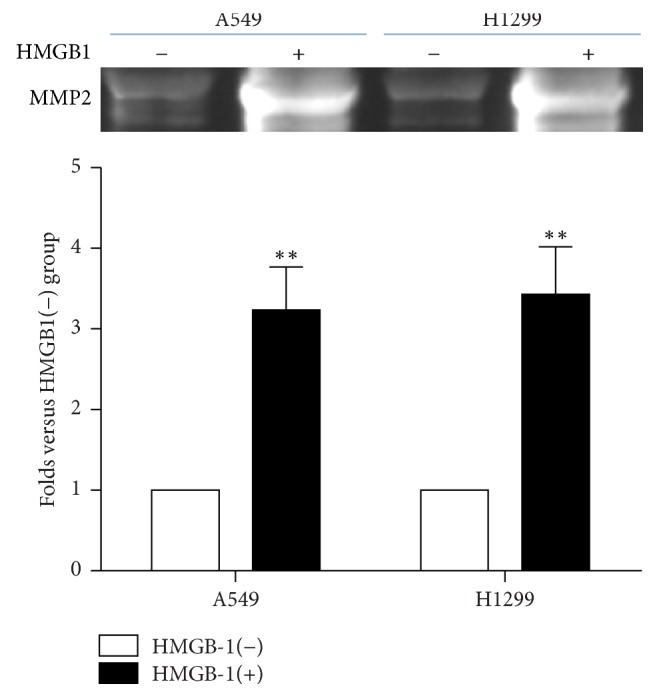
HMGB1 promoted the activity of MMP2 in lung cancer cells. Pretreatment of HMGB1 significantly promoted the activity of MMP2 in A549 and H1299 cells (^*∗∗*^*P* < 0.01 compared with the control). Significant differences were assessed by the Student *t*-test.

To further study the mechanism of HMGB1-promoting lung cancer cell invasion, gelatin zymography assays were performed in lung cancer cell lines, A549 and H1299, after pretreatment with HMGB1. The results showed that MMP2 enzyme activities in the A549 and H1299 cells were significantly higher after treatment with HMGB1 than the control group (Figure 3).

### 3.6. HMGB1 Promotes MMP-2 Expression via the NF-*κ*B Pathway

**Figure 4:**
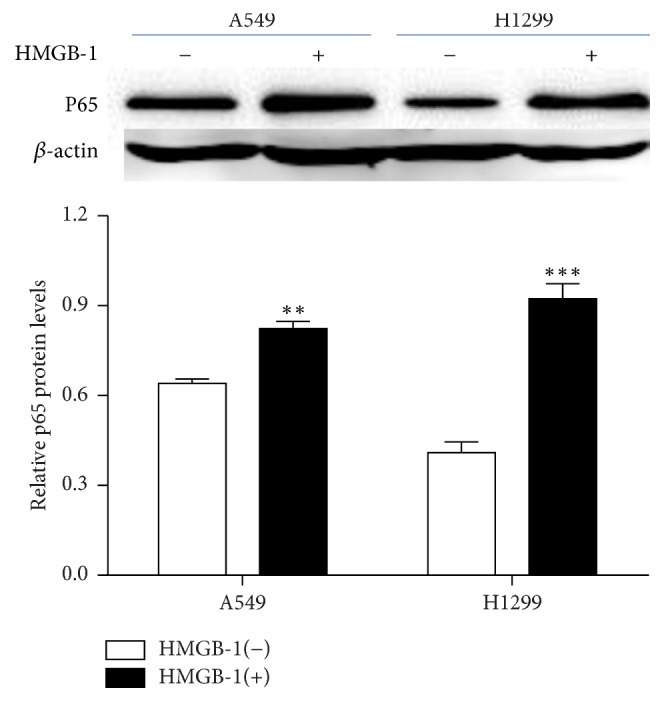
Western blot analysis of the expression level of p65 in lung cancer cells after treatment with HMGB1. Pretreatment of HMGB1 significantly increased the expression level of p65 in A549 and H1299 cells. (^*∗∗*^*P* < 0.01, ^*∗∗∗*^*P* < 0.001 compared with the control). Significant differences were assessed by the Student *t*-test.

**Figure 5:**
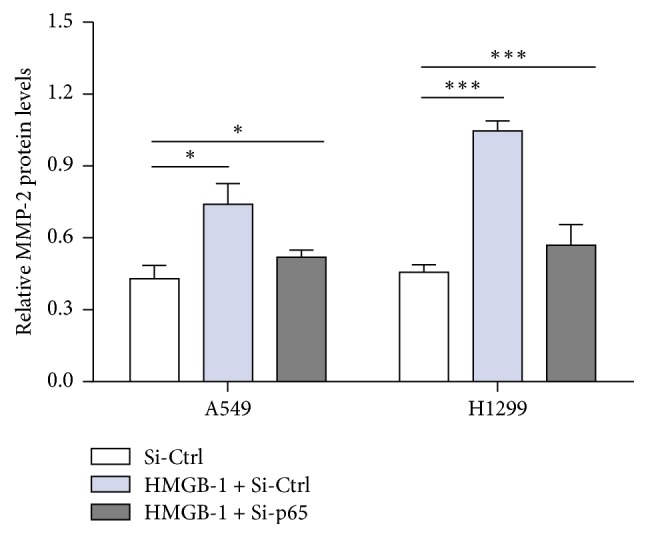
Western blot analysis of the expression level of MMP2 in lung cancer cells after SiRNA transfection. Pretreatment of HMGB1 significantly increased the expression level of MMP2 in A549 and H1299 cells. However, inhibition of p65 significantly attenuated the expression level of MMP2 in A549 and H1299 cells. (^*∗*^*P* < 0.05, ^*∗∗∗*^*P* < 0.001 compared with the control). Significant differences were assessed by the Student *t*-test.

Our previous study showed that HMGB1 induced NF-*κ*B expression in human bronchial epithelial cells in a dose-dependent manner. To investigate the mechanism of HMGB1 promotion of MMP2 expression, western blots were used to detect NF-*κ*B expression. The results showed significantly increased NF-*κ*B expression of the HMGB1 pretreatment group versus the control group (Figure 4). In addition, p65 siRNA was transfected into the A549 and H1299 lung cancer cell lines, together with HMGB1 treatment. Compared with the control group, treatment with HMGB1 promoted MMP2 expression when there was no p65 siRNA transfection, while the expression of MMP2 was significantly decreased in the p65 siRNA transfection groups compared with the p65 siRNA control group (Figure 5). These results showed that HMGB1 increased the expression of MMP2, thus promoting the invasive ability of lung cancer cells via the NF-*κ*B pathway.

## 4. Discussion

Little is known about the pathogenesis of NSCLC and the identification of effective markers for early diagnosis and prognosis of NSCLC. Therefore, identification of effective markers for early diagnosis and prognosis of NSCLC has become an important objective of recent studies. HMGB1 has been reported to be associated with the progression of NSCLC. Recent studies have shown that HMGB1 can participate in cell differentiation, migration, regeneration, and mediation of inflammation, particularly in binding to RAGE, to affect tumor growth, invasion, and metastasis [38]. Shang et al. first reported that serum HMGB1 levels were significantly increased in patients with lung cancer when compared with control subjects [20]. In the present study, we used tumor tissue microarrays to characterize an independent cohort of 90 NSCLC patients for the expression of HMGB1. The clinical analyses showed a significantly higher expression of HMGB1 in the lung cancer tissues than in the adjacent normal tissues. Furthermore, there was a correlation between HMGB1 expression with TNM staging and the postoperative survival of lung cancer patients, which was similar to the results of previous studies [17]. Taken together, the results suggested that HMGB1 plays an important role in tumor progression and may be useful in the prognoses of NSCLC patients.

It is well-known that tumor cell migration and invasion are major factors leading to cancer deterioration that affects the prognoses of patients. Many studies have reported that HMGB1 is associated with cancer cell migration and invasion. Xiao and Liu suggested that silencing of HMGB1 inhibited lung cancer migration and invasion [39]. Consistent with the above studies, the Transwell results in the present study showed that pretreatment with HMGB1 improved the invasiveness of lung cancer cells.

MMPs are important in angiogenesis, growth, and metastasis of tumors [40]. As a MMP superfamily member, MMP2 is localized in a proteolytically active form on the surface of invasive cells, which has been found to play an important role in tumor invasion and metastasis [41, 42]. Activating MMP2 generates type IV collagenase, which can degrade the proteins of the extracellular matrix of tumor cells, leading to tumor cell invasion and metastasis [43].

The p65 preprotein, a transcription factor involved in cellular signal transduction, is associated with the activity of transcription factors via various mechanisms [19, 43, 44]. Previous reports suggested that the interaction between HMGB1 and RAGE triggered the activation of NF-*κ*B, MAPK, and MMP-2/MMP-9 signaling pathways, which are associated with tumor growth, invasion, and metastasis [20, 26, 45–47]. Fujioka et al. reported a positive correlation between the transcription factor p65 and tumor metastasis [48]. Furthermore, it has been confirmed that NF-*κ*B can activate the MMP2 promoter and activate the membrane type protease (MT1 MMP), which further leads to hydrolysis and activation of MMP2 [49, 50]. Importantly, our study showed that HMGB1 specifically promoted MMP2 and NF-*κ*B expression. However, p65 siRNA treatment significantly reduced MMP2 expression, suggesting that HMGB1 accelerated the MMP2 expression via the NF-*κ*B pathway to promote lung cancer migration and invasion.

Taken together, our studies showed that the high expression of HMGB1 in lung cancer, which may be used in the prognoses of lung cancer patients, promoted lung cancer invasion and metastasis by upregulating the expression and activity of MMP-2 via an NF-*κ*B-dependent pathway. These findings may assist in clinical diagnoses and suggest therapeutic strategies for patients with NSCLC.

## Figures and Tables

**Table 1:** HMGB1 expression level in lung cancer tissues and normal tissues adjacent to lung cancers.

Variables	HMGB1 staining			
	Low (%)	High (%)	Total	*P* ^*∗*^
Lung cancer tissues	39 (43.3)	51 (56.7)	90	0.001
Adjacent noncancerous tissues	63 (70.0)	27 (30.0)	90	

^*∗*^The Student *t*-test for *P* value.

**Table 2:** The relationship between high mobility group box protein 1 staining and the clinicopathological characteristics of 90 lung cancer patients.

Variables	HMGB-1 staining			
	Low (%)	High (%)	Total	*P* ^*∗*^
*Age*				
≥60 years	23 (41.8)	32 (58.2)	55	0.828
<60 years	16 (45.7)	19 (54.3)	35	
*Gender*				
Male	19 (38.8)	30 (61.2)	49	0.396
Female	20 (48.8)	21 (51.2)	41	
*Tumor size*				
≥4 cm	23 (44.2)	28 (55.8)	51	0.832
<4 cm	16 (41.0)	23 (59.0)	39	
*pT status*				
pT_1_-pT_2_	34 (50.7)	33 (49.3)	67	0.027
pT_3_-pT_4_	5 (21.7)	18 (78.3)	23	
*pN status*				
pN_0_	22(56.4)	17(43.6)	39	0.019
pN_1_–pN_3_	10(27.8)	26(72.2)	36	
*TNM stage*				
I-II	35 (50.7)	34 (49.3)	69	0.012
III	4 (19.0)	17 (81.0)	21	

^*∗*^The Student *t*-test for *P* value.
